# NLRP3 inflammasome inhibition attenuates sepsis-induced platelet activation and prevents multi-organ injury in cecal-ligation puncture

**DOI:** 10.1371/journal.pone.0234039

**Published:** 2020-06-17

**Authors:** Denise C. Cornelius, Olivia K. Travis, Robert W. Tramel, Marivee Borges-Rodriguez, Cedar H. Baik, Mallory Greer, Chelsea A. Giachelli, Geilda A. Tardo, Jan M. Williams

**Affiliations:** 1 Department of Emergency Medicine, University of Mississippi Medical Center, Jackson, Mississippi, United States of America; 2 Department of Pharmacology & Toxicology, University of Mississippi Medical Center, Jackson, Mississippi, United States of America; 3 Cardiovascular-Renal Research Center, University of Mississippi Medical Center, Jackson, Mississippi, United States of America; 4 Department of Pediatrics, University of Mississippi Medical Center, Jackson, Mississippi, United States of America; University of the Pacific, UNITED STATES

## Abstract

Sepsis is characterized by organ dysfunction due to a dysregulated immune response to infection. Currently, no effective treatment for sepsis exists. Platelets are recognized as mediators of the immune response and may be a potential therapeutic target for the treatment of sepsis. We previously demonstrated that NLRP3 inflammasome activation in sepsis-induced activated platelets was associated with multi-organ injury in the cecal-ligation puncture (CLP) rat model of sepsis. In this study, we tested the hypothesis that inhibition of NLRP3 would inhibit platelet activation and attenuate multi-organ injury in the CLP rat. CLP (n = 10) or Sham (n = 10) surgery were performed in male and female Sprague-Dawley rats. A subset of CLP rats were treated with MCC950 (50mg/kg/d), a specific NLRP3 inhibitor (CLP+MCC950, n = 10). At 72 hrs. post-CLP, blood and organs were harvested for analysis of platelet activation, NLRP3 activation, inflammation and end organ damage. Platelet activation increased from 8±0.8% in Sham to 16±1% in CLP, and was reduced to 9±1% in CLP+M rats (p<0.05). NLRP3 activation was also increased in platelets of CLP vs Sham. NLRP3 expression was unchanged in kidney and lung after CLP, but Caspase 1 expression and IL-1β were increased. MCC950 treatment attenuated NLRP3 activation in platelets. Plasma, kidney, and lung levels of NLRP3 inflammasome associated cytokines, IL-1ß and IL-18, were significantly increased in CLP compared to Sham rats. Inhibition of NLRP3 normalized cytokine levels. Glomerular injury, pulmonary edema, and endothelial dysfunction markers were increased in CLP rats vs Sham. MCC950 treatment significantly decreased renal and pulmonary injury and endothelial dysfunction in CLP+M. Our results demonstrate a role for NLRP3 in contributing to platelet activation and multi-organ injury in sepsis.

## Introduction

Sepsis is a life-threatening syndrome of organ dysfunction caused by an exaggerated host immune response to infection [1]. The development of acute lung injury (ALI) and/or acute kidney injury (AKI) are the most common complications of sepsis and are associated with increased mortality in sepsis patients [2, 3]. The highest incidence of ALI occurs in patients with sepsis [4]. Moreover, sepsis accounts for 50% of reported cases of AKI in developed countries [5–7]. Forty-three percent of sepsis-associated deaths are caused by multiple organ failure [8]. There is currently no effective treatment to cure sepsis. Rather, standard of care focuses on eliminating the nexus of infection, controlling inflammation, and treating organ dysfunction [9]. Because of the dysregulated immune response that leads to organ injury, trials to identify novel therapies have targeted inflammatory cytokines. However, therapies, targeting a single component of the inflammatory cascade have failed to reduce the multiple organ injury/dysfunction or mortality rate associated with sepsis [10]. It is therefore, imperative to identity novel therapeutic targets for treatment of sepsis with more effective clinical impact.

The NOD-like receptor protein 3 inflammasome (NLRP3) is a cytoplasmic complex responsible for activation of IL-1β and IL-18 (pro-inflammatory cytokines) and the pro-inflammatory cell death known as pyroptosis [11]. Activation of the NLRP3 inflammasome stimulates a number of pro-inflammatory pathways and mechanisms that lead to the production of additional pro-inflammatory cytokines and activation of the innate and adaptive immune responses. NLRP3 activation is linked to a number of inflammatory conditions, including sepsis [12] and several studies have shown that NLRP3 null animals are protected against sepsis induced organ injury [13–17] and shock [15, 18]. However, the mechanisms by which this occurs have not been fully elucidated.

Septic patients with increased platelet activation and low platelet count are prone to develop multiple organ dysfunction and have increased 90-day mortality [1, 19–22]. The contribution of platelets to sepsis progression goes beyond thrombosis and coagulation. Platelets have emerged as major drivers of the innate and adaptive immune response. We have recently shown that NLRP3 inflammasome activation occurs in platelets and is associated with renal glomerular injury and pulmonary edema in the cecal-ligation and puncture (CLP) rat model of sepsis [23]. We also demonstrated that NLRP3 inflammasome assembly and activation was increased in platelets activated by LPS treatment *in vitro*, or CLP stimulation *in vivo* [23]. However, that study did not determine if NLRP3 activation caused platelet activation or vice versa [23]. In the present study, we hypothesized that inhibition of NLRP3 with a specific inhibitor would attenuate platelet activation and inflammation in a 72-hr CLP rat model. We further hypothesized that inhibition of NLRP3 would improve renal function and prevent immune cell infiltration into the lungs to attenuate multi-organ injury induced by polymicrobial sepsis.

## Materials and methods

### Animals

Male and female Sprague-Dawley rats purchased from Envigo (Indianapolis, IN) were used in this study. All experimental procedures executed in this study were in accordance with the National Institutes of Health guidelines for use and care of animals. All protocols were approved by the Institutional Animal Care and Use Committee at the University of Mississippi Medical Center. The care and handling of the animals were in accord with the National Institutes of Health guidelines for ethical animal treatment.

### Cecal ligation and puncture and NLRP3 inhibition *in vivo*

All of our *in vivo* experiments were performed in 12–13 week old male rats weighing approximately 300–325 g. The animals were randomly divided into 2 groups: sham operation group (Sham, n = 12) and cecal ligation puncture group (CLP, n = 12). Under isoflurane anesthesia the CLP surgery was performed on a subset of rats to induce abdominal polymicrobial sepsis as previously described [24]. Preheated saline (20 ml/kg, 37°C) was subcutaneously injected immediately after the operation for resuscitation. Sham-operated animals underwent the same surgical procedure without cecum ligation or puncture. The rats were placed back into their cages after surgery, and food and water were provided ad libitum. At 24 hours post-CLP, the prior incision was re-opened and the necrotic cecum was carefully excised. The abdominal cavity was washed twice with 30 mL of warm sterile saline solution. The abdominal incision was again closed in layers. Animals were administered a broad-spectrum antibiotic daily Naxcel (5 mg/kg) daily and buprenorphine SR (1.2 mg/kg) to control pain over the 72 hr. experimental period. The NLRP3 inflammasome was inhibited in a subset of CLP rats (n = 12) by daily i.p. administration of the specific NLRP3 inhibitor, MCC950 (50 mg/kg/day) [25, 26] for 3 days. At the end of the protocol, the animals were sacrificed by thoracotomy and removal of the heart under deep anesthesia. Blood and tissues were collected for further analysis.

### Platelet isolation

Arterial blood was drawn into acid-citrate-dextrose vacutainer tubes and centrifuged at 200*g* for 20 minutes to obtain platelet-rich plasma. Platelet rich plasma (PRP) was mixed with an equal volume of HEP buffer (140 mM NaCl, 2.7 mM KCl, 3.8 mM HEPES, 5 mM EGTA, pH 7.4) containing 1 μM PGE1. This mixture was then centrifuged at 100*g* for 20 minutes to pellet remaining RBC and WBC in which40 μL of the supernatant were used to count platelets. The PRP was then centrifuged at 800*g* for 20 minutes, the supernatant was discarded and the pellet was resuspended in Tyrode’s buffer (134mM NaCl, 12mM NaHCO_3_, 2.9mM KCl, 0.34mM Na_2_HPO_4_, 1mM MgCl_2_, 10mM HEPES) containing 5mM glucose and 3mg/mL BSA. Additionally, 5x10^5^ platelets from CLP and SHAM rats were plated in 3 wells of a 96-well plate in a total of 200μL of Tyrode’s buffer. After overnight incubation at 37°C, the media was collected and pooled for further analysis.

### *In vitro* LPS stimulation

1x10^7^ platelets from normal Sprague Dawley rats were plated in the well of a 96-well plate and treated with 100 ng/mL *E*. *coli* LPS (L5024, Millipore Sigma, St. Louis, MO), 100 ng/mL recombinant LPS binding protein (6635-LP-025, R&D Systems, Minneapolis, MN), and 100 ng/mL recombinant CD14 (SRP6036, Millipore Sigma) or Tyrode’s buffer only with or without MCC950 for 2 hours at 37°C. To inhibit NLRP3 activation, subsets of platelets were treated with 1 μg, 5μg, or 10 μg MCC950. After incubation, the media was collected and pooled for measurement of IL-1β secretion. Six separate experiments were performed.

### Flow cytometry

Freshly isolated platelets (10^6^) were resuspended in 50 μL of Tyrode’s buffer. Platelets were incubated (RT for 30 minutes) with fluorescein isothocyanate (FITC) -conjugated anti-mouse CD41 (Clone MWReg30, BioLegend, San Diego, CA) and Allophycocyanin (APC) conjugated anti-mouse/rat CD62P (Clone RMP-1, Biolegend). A minimum of 10,000 events per gate were acquired using a MACsQuant Analyzer 10 (Miltenyi Biotec, Auburn, CA) and analyzed using FlowLogic software (Innovai, Sydney, Australia). Platelets were distinguished by specific binding of anti-CD41 and characteristic forward and side scattering. Platelets staining positive for CD41 and CD62P were designated as activated platelets.

### Confocal and light microscopy

Platelets (10^7^) from Sham or CLP rats or stimulated with or without LPS were allowed to adhere to permanox slides (Lab-Tek, Thermo Fisher Scientific, Waltham, MA). Adhered cells were fixed in 10% formalin for 30 minutes, washed 3Xs with PBS for 10 minutes at RT, and permeabilized with ice-cold 100% methanol for 10 minutes. The slides were blocked in PBS with 10% goat and rabbit serum for 1 hr., washed and incubated with rabbit anti-NLRP3 antibody (NBP2-12446, Novus Biologicals, Littleton, CO) for 2 hours at RT. Slides were then washed and incubated with anti-rabbit Alexa Fluor 488 (A11070, Thermo Fisher Scientific) for 1 hour at RT. The slides were washed again and incubated with Alexa Fluor 647-conjugated rabbit anti-ASC antibody (NBP1-78977AF647, Novus Biologicals) for 1 hour at room temperature. Controls were processed identically, except for omission of the primary antibodies. Preparations were mounted in Cytoseal 60 (Thermo Scientific) and analyzed on a Nikon C1 (Nikon) confocal scanning microscope. The images were captured using a Nikon Eclipse 55i microscope equipped with a Nikon DS-Fi1 color camera (Nikon, Melville, NY).

#### Caspase 1 activity assay

Caspase 1 activity was assessed in isolated platelets from each group using the Caspase 1 Assay Kit (Abcam, Cambridge, MA) according to the manufacturer’s instructions.

### Quantification of biomarkers of endothelial permeability and cytokines

Plasma levels of Angiopoietin-2 (MANG20, R&D Systems) and Endocan-1 (MBS039900, MyBioSource, San Diego, CA) were measured as markers of endothelial permeability by ELISA according to the manufacturer’s protocol. Plasma, kidney, and lung IL-1β (RLB00, R&D Systems) and IL-18 (ab213909, Abcam, Cambridge, MA) were quantified by ELISA according to the manufacturer’s protocol. Kidneys and lung lysates were prepared with the BioPlex Cell Lysis Kit (171304011, BioRad, Hercules, CA) according to the manufacturer’s protocol. Plasma levels of IL-6, IL-17, and IL-10 were quantified by luminex multi-bead technology (Bio-Rad). Plasma levels of Platelet Factor 4 (a marker of platelet activation) were measured by ELISA (MCX400, RnD Systems). Protein was quantified by BCA analysis in kidney and lung lysates and culture media. IL-1β was quantified in platelet culture media via ELISA. Data from kidney and lung lysates, and culture media were normalized to protein.

### Western blot

Kidney and lung lysates were prepared for Western blot using the BioPlex Cell Lysis Kit (BioRad) according to the manufacturer’s protocol. A total of 100 μg of protein were separated by SDS-PAGE using a polyacrylamide gel (4–15%). The proteins were transferred onto 0.2 μm nitrocellulose membranes (BioRad). Membranes were blocked with blocking buffer (0.5% non-fat milk in 1x TBST) for 1 hour at room temperature and incubated overnight at 4°C with primary antibody directed against NLRP3 (1:1000; Recombinant anti-NLRP3 antibody, ab264468, Abcam) or Caspase-1 (1:1000; Recombinant anti-pro Caspase-1 + p10 + p12 antibody, ab238972, Abcam). After washing, the membranes were incubated with HRP-conjugated secondary antibody (1:2500; Goat anti-rabbit IgG H&L, ab205718, Abcam) for 1 hr at room temperature.The protein bands were developed with enhanced chemiluminescence reagents (BioRad) and visualized on a BioRad Chemidoc. The intensity of specific bands were quantified by densitometry using Image J (National Institutes of Health, Washington, DC) and expression was normalized with respect to β-actin expression (1:2000; Anti-β-actin antibody, a1978-100ul, Millipore Sigma, St. Louis, MO). Secondary antibody to β-actin: 1:2000; Goat anti-mouse IgG (H +L)-HRP conjugate, 1706516, BioRad.

### Assessment of lung injury

Histological evaluation was performed on lungs via hematoxylin and eosin (H&E) staining. A lung lobe was formalin-fixed. The tissue was subsequently paraffin-embedded and 5-μm sections were stained with (H&E). Sections were evaluated for neutrophil infiltration into the alveolar space. Five sections were scored in a blinded fashion on a scale of 0–2 with 0 representing o neutrophils in the alveolar space, 1 representing 1–5 neutrophils in the alveolar space, and 2 representing >5 neutrophils in the alveolar space. Images were captured using a Nikon Eclipse 55i microscope equipped with a Nikon DS-Fi1 color camera (Nikon Inc.; Melville, NY) at 40X using NIS-Elements D 3.0 software. To quantify the magnitude of pulmonary edema, a lung wet-to-dry (W/D) ratio was determined. The wet weight was determined by excising the left lung lobe, blotting it dry, and then immediately weighing it. The excised lung lobe was then placed in an oven at 80°C for 72 hrs. and then re-weighed to obtain the dry weight. The W/D ratio was calculated by dividing the wet weight by the dry weight.

### Assessment of renal glomerular injury

Kidneys were collected, weighed and fixed in a 10% buffered formalin solution. Paraffin sections (3μm) were prepared and stained with Periodic acid-Schiff to assess the degree of glomerular injury on approximately 30 images per section per rat. Thirty glomeruli per section were scored in a blinded fashion on a 0–4 scale with 0 representing a normal glomerulus, 1 representing a 25% of loss, 2 representing a 50% loss, 3 representing a 75% loss, and 4 representing >75% loss of capillaries in the tuft. Images were captured using the same microscope, camera, and software as mentioned previously.

### Measurement of renal function

During the CLP procedure, a catheter was inserted into the jugular vein and exteriorized subcutaneously at the back of the neck. The catheter was flushed with heparinized saline each day until the end of the three-day protocol. On the last day of the protocol, rats underwent brief isoflurane anesthesia in order to assemble the NIC-Kidney device (MediBeacon; Mannheim, Germany) made of two light emitting diodes that excite FITC-sinistrin at 480 nm, a photodiode that emits light at 531 nm, a microprocessor, and a battery. This device was attached to the back of the rat using a double-sided adhesive patch (MediBeacon; Mannheim, Germany) and secured with a rodent jacket to a shaved region on the back of the rat. Rats were allowed to recover in separate cages for 15 minutes and a baseline measurement was recorded. After baseline measurements, a bolus injection of FITC-sinistrin (5mg/100g body weight; FTC-FS001 MediBeacon; Mannheim, Germany) was administered via the jugular vein followed by a bolus injection of sterile saline. During a two-hour period after the bolus injection, excretion kinetics of FITC-sinistrin were measured transcutaneously and was used to calculate elimination half-life (t_1/2_) of FITC-sinistrin using a one compartment model with the MDPLab evaluation software (MediBeacon; Mannheim, Germany) to calculate glomerular filtration rate (GFR) from a validated empirically-derived conversion factor [27–29].

### Statistical analysis

All data are expressed as mean ± SEM. All data were checked to be consistent with Gaussian distribution by D’Agostino-Pearson normality test. Statistical analyses were performed with one-way ANOVA with Tukey’s multiple comparisons test as post hoc analysis. A value of *p*<0.05 was considered statistically significant.

## Results

### Lung and kidney NLRP3 and Caspase 1 expression

**Fig 1:**
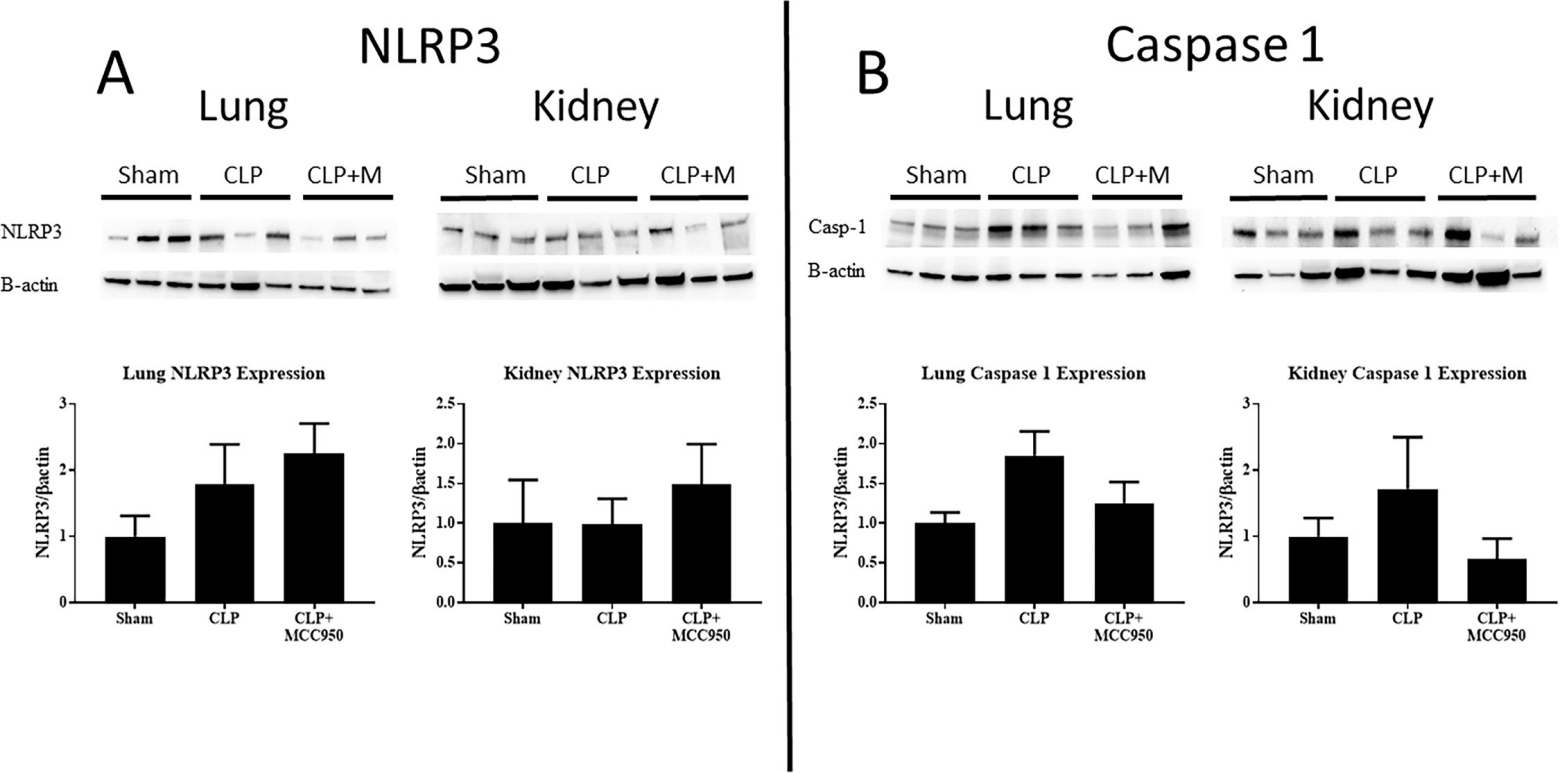
NLRP3 and Caspase 1 protein. NLRP3 (A) and Caspase 1 (B) protein levels were quantified in kidney and lung via western blot and normalized to β-actin. NLRP3 (A) and Caspase 1 (B) expression in lungs and kidneys in response to sepsis and after treatment with the NLRP3 specific inhibitor, MCC950 (n = 5-6/group). *p<0.05 vs Sham, #p<0.05 vs CLP.

Pulmonary and renal expression of NLRP3 and Caspase 1 were evaluated via Western Blot (Fig 1) in Sham, CLP, and CLP+MCC950 rats. We observed that NLRP3 expression in the lung nearly doubled after CLP, a difference that was not significant. MCC950 administration did not alter lung NLRP3 protein levels in CLP rats (Fig 1A). Expression of Caspase 1 in the lung trended towards a significant increase in CLP vs Sham rats (p = 0.06). Treatment with the NLRP3 inhibitor did not significantly alter caspase 1 expression in the lung (Fig 1B). Renal NLRP3 and Caspase 1 expression were also unchanged in all groups (Fig 1).

### Platelet NLRP3 activation and Caspase 1 activity

**Fig 2:**
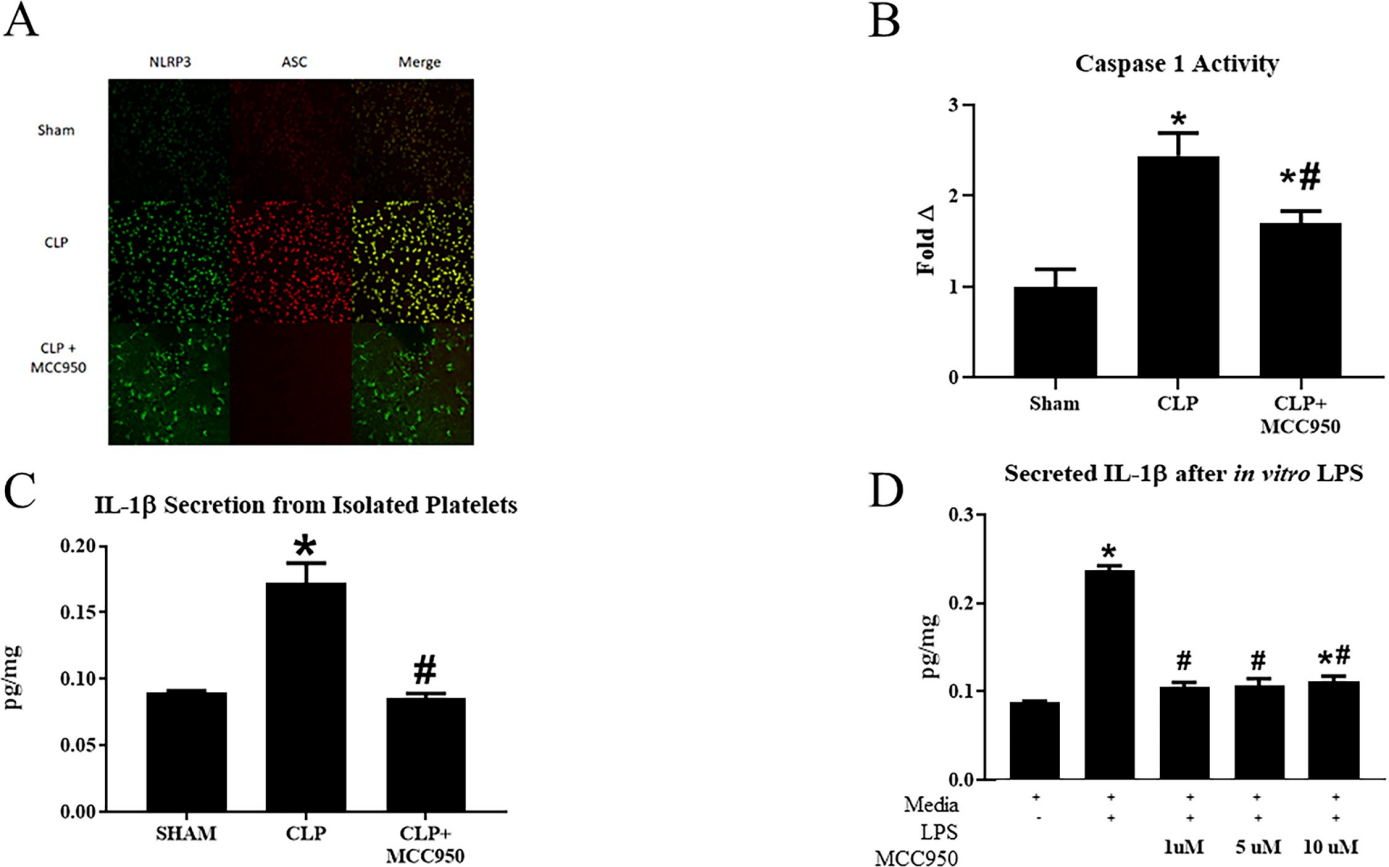
NLRP3 activation in platelets. NLRP3 activation was evaluated by visualizing the co-localization of NLRP3 and apoptosis-associated speck-like protein (ASC) using immunocytochemistry (**A**). Caspase 1 activity was assessed in all groups (n = 6/group, **B**). Platelets were isolated from Sham, CLP, and CLP+MCC950 animals and cultured in media overnight and IL-1β secretion was measured via ELISA (**C**). Platelets were stimulated *in vitro* with LPS and cultured with or without MCC950 at varying doses (1, 5, or 10 μM). Media was collected and IL-1β was measured in media via ELISA (**D**). *P* < 0.05 versus Sham *p<0.05 vs Sham or media only, #p<0.05 vs CLP or LPS-stimulated platelets.

NLRP3 activation in platelets was evaluated by visualizing the co-localization of NLRP3 with apoptosis-associated speck-like protein (ASC) using immunocytochemistry [30–32] and assessment of caspase 1 activity. As we have previously demonstrated, CLP platelets demonstrate increased co-localization of inflammasome components compared to Sham platelets. Importantly, platelets from MCC950 treated CLP rats demonstrated decreased inflammasome assembly (Fig 2A). Caspase-1 was evaluated in platelets isolated from Sham, CLP, and CLP+MCC950 rats. Caspase 1 activity was significantly increased in platelets isolated from CLP rats compared to platelets isolated from Sham rats. Caspase 1 activity increased by more than 2-fold in CLP platelets compared to Sham platelets (p<0.05, Fig 2B). Caspase-1 activity in platelets was significantly reduced in CLP animals treated with the NLRP3 inhibitor (p<0.05 vs CLP). Release of IL-1β into culture media by platelets isolated from animals in each group was quantified by ELISA. IL-1β secreted in culture media from Sham platelets (0.089 pg/mg) was less than that secreted by CLP platelets (0.17 pg/mg, p<0.05 vs Sham, Fig 2C). Platelets from CLP+MCC950 animals secreted significantly less IL-1β (0.085pg/mg) compared to CLP platelets (Fig 2C, p>0.05). Treatment of *in vitro* stimulated *LPS-*activated platelets with MCC950 also inhibited IL-1β secretion by platelets. LPS-activated platelets secreted significantly more IL-1β compared to non-activated platelets (0.24 pg/mg vs 0.088 pg/mg, p<0.05). Treatment of LPS-activated platelets with 1, 5, or 10 μM MCC950 significantly decreased secretion of IL-1β (Fig 2D).

### Platelet activation

**Fig 3:**
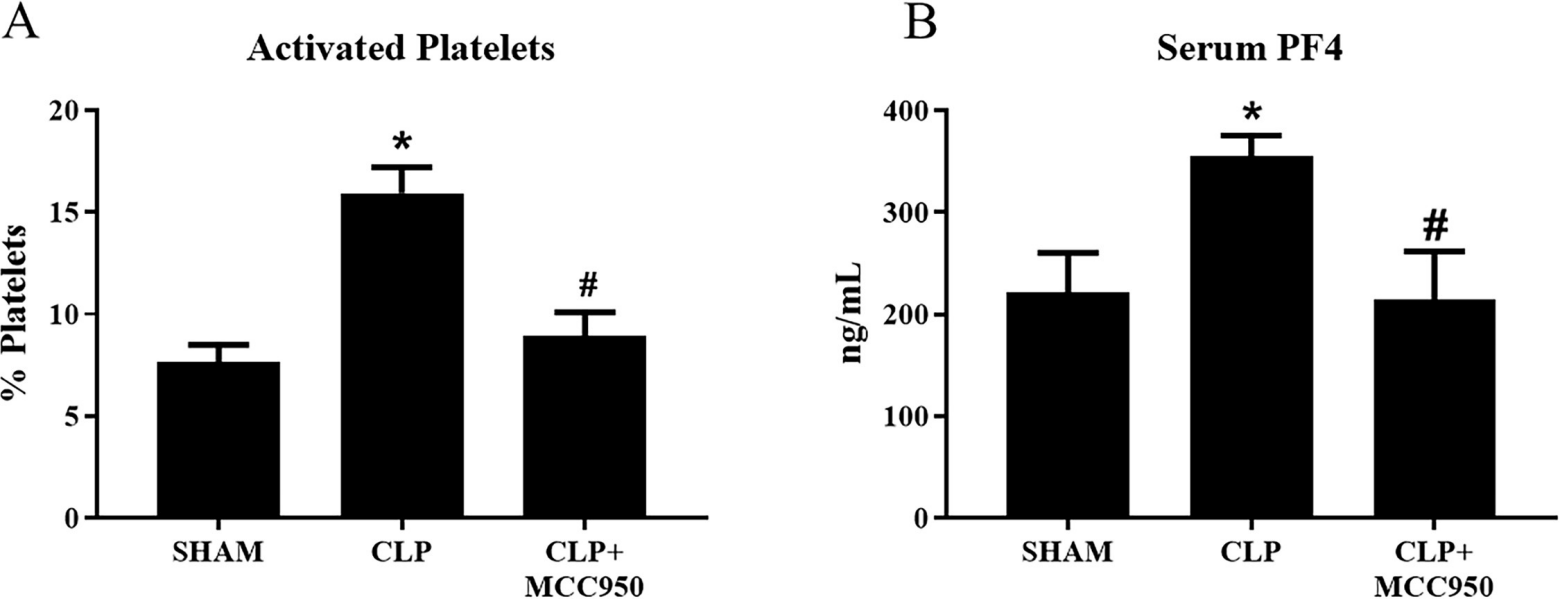
Platelet activation. Platelets were isolated from Sham, CLP, and CLP+MCC950 (n = 10/group) and assessed for activation via flow cytometry. Serum platelet factor 4 (PF$) was assessed via ELISA (n = 10/group) *p<0.05 vs Sham, #p<0.05 vs CLP.

Isolated platelets from Sham, CLP, and CLP+MCC950 rats were assessed for activation via flow cytometry. Activated platelets significantly increased from 8±0.8% in Sham rats to 16±1% in CLP rats, and this was completely normalized to 9±1% in CLP+MCC950 rats (p< 0.05, Fig 3A). Serum Platelet Factor4 (PF4) levels were also measured as a marker of platelet activation. Serum PF4 increased from 221.9±38.5 ng/mL in Sham to 355.2±20.46 ng/mL in CLP, and significantly decreased to 214.5±47.44 ng/mL in CLP+MCC950 (p<0.05, Fig 3B)

### Inflammasome-associated cytokines and systemic inflammation

**Fig 4:**
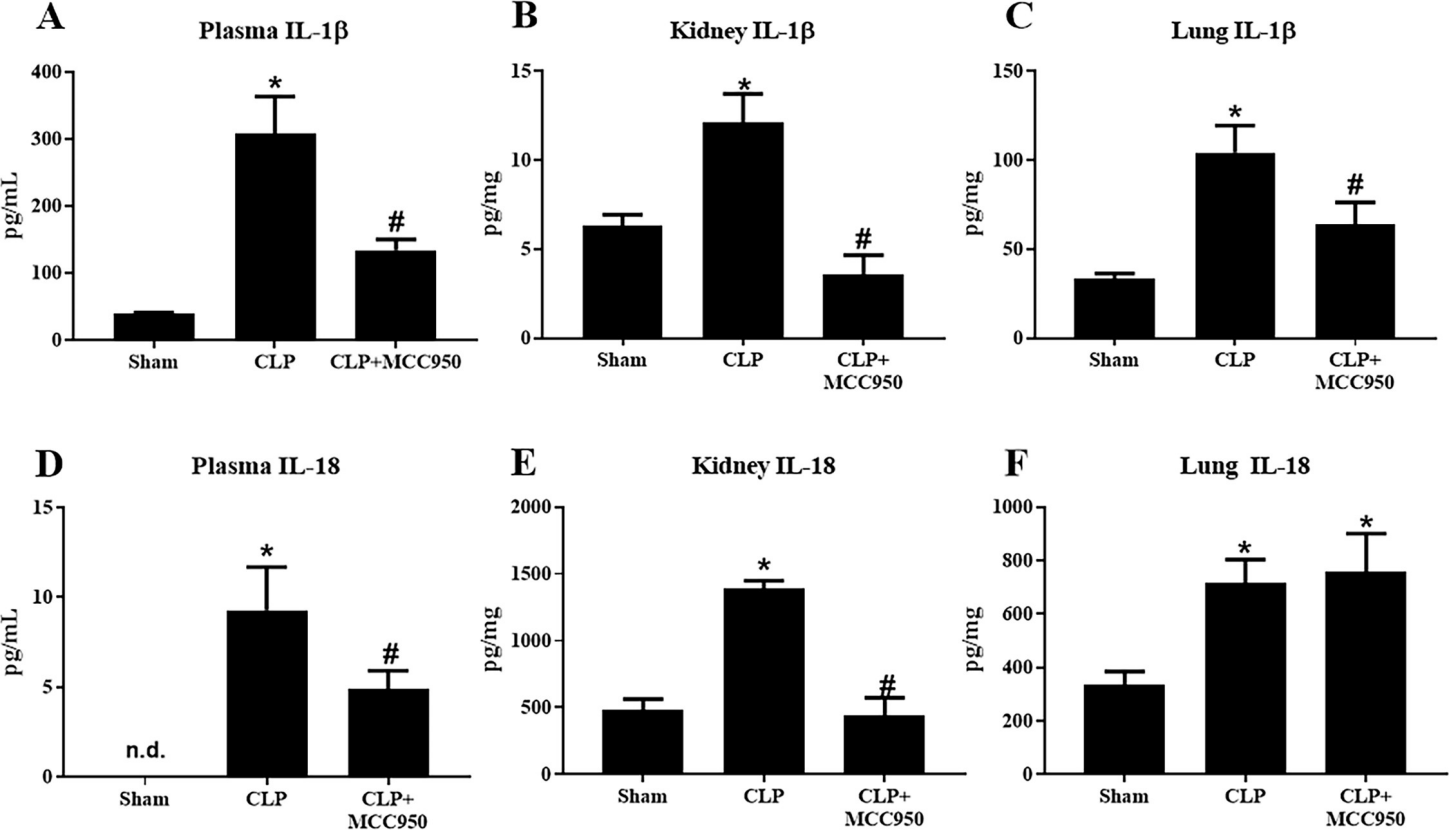
Inflammasome-associated cytokines. IL‐1*β* and IL‐18 protein levels in response to CLP. Plasma (A and D), kidney (B and E), and lung (C and F) IL‐1*β* and IL‐18 were measured via ELISA in Sham, CLP, and CLP rats treated with MCC950 (*n* = 12/group). *P < 0.05 versus Sham *p<0.05 vs Sham, #p<0.05 vs CLP.

Caspase-1 catalyzes the cleavage of IL-1β and IL-18 into their active forms, thus we measured the levels these cytokines in the plasma, lungs, and kidneys of Sham, CLP, and CLP+MCC950 rats via ELISA. Plasma IL-1β significantly increased from 39±2 pg/mL in Sham to 307±56 pg/mL in CLP, and this was significantly reduced to 134±16 pg/mL in CLP+MCC950 (p<0.05, Fig 4A). Renal IL-1β followed a similar pattern with a significant increase from 6±0.6 pg/mg in Sham to 12±2 pg/mg in CLP and complete normalization to 4±1 pg/mg in CLP+MCC950 (p<0.05, Fig 4B). Finally, pulmonary IL-1β significantly increased from 33±3 pg/mg in Sham to 105±15 pg/mg in CLP and significantly decreased to 64±13 pg/mg in CLP+MCC950 (p<0.05, Fig 4C).

Plasma IL-18 was undetectable in Sham rats, but significantly higher at 9.3±2.4 pg/mL in CLP rats (p<0.05). MCC950 administration significantly decreased the levels to 4.9±1.0 pg/mL (p<0.05, Fig 4D). Similarly, renal IL-18 significantly increased from 480±84 pg/mg in Sham to 1394±54 pg/mg in CLP and was normalized to 441±132 pg/mg in CLP+MCC950 (p<0.05, Fig 4E). In the lung, IL-18 significantly increased from 336±48 pg/mg in Sham to 718±85 pg/mg in CLP; however, the levels remained elevated in CLP+MCC950 rats (756±144 pg/mg) (p<0.05 vs Sham, Fig 4F).

Plasma levels of the pro-inflammatory cytokines IL-6 and IL-17, and the anti-inflammatory cytokine IL-10 were measured in animals from each group (Table 1). Plasma IL-6 doubled in CLP vs Sham. Treatment with MCC950 did not alter IL-6 plasma levels in CLP animals. The pro-inflammatory cytokine IL-17, was significantly increased in CLP animals compared to Sham control, and was normalized after MCC950 treatment (p<0.05 vs CLP. Conversely, IL-10, a potent anti-inflammatory cytokine, was significantly less in CLP vs Sham. Inhibition of NLRP3 activation resulted in increased plasma IL-10 in treated CLPs (p<0.05 vs CLP).

**Table 1:** Pro and anti-inflammatory cytokines.

Cytokine	Sham	CLP	CLP+MCC950
IL-6	5.04±1.35	10.97±3.31	8.94±1.35
IL-17	1.09±0.26	2.8±0.87*	0.84±0.23^#^
IL-10	24.31±4.13	11.79±1.27*	24.91±2.69^#^

**Plasma Cytokines** Plasma levels ofIL-6, IL-17, and IL-10 were measured in each group by multi-bead luminex assay. n = 9-10/group; *p>0.05 vs Sham; #p<0.054 vs CLP

### Endothelial permeability

**Fig 5:**
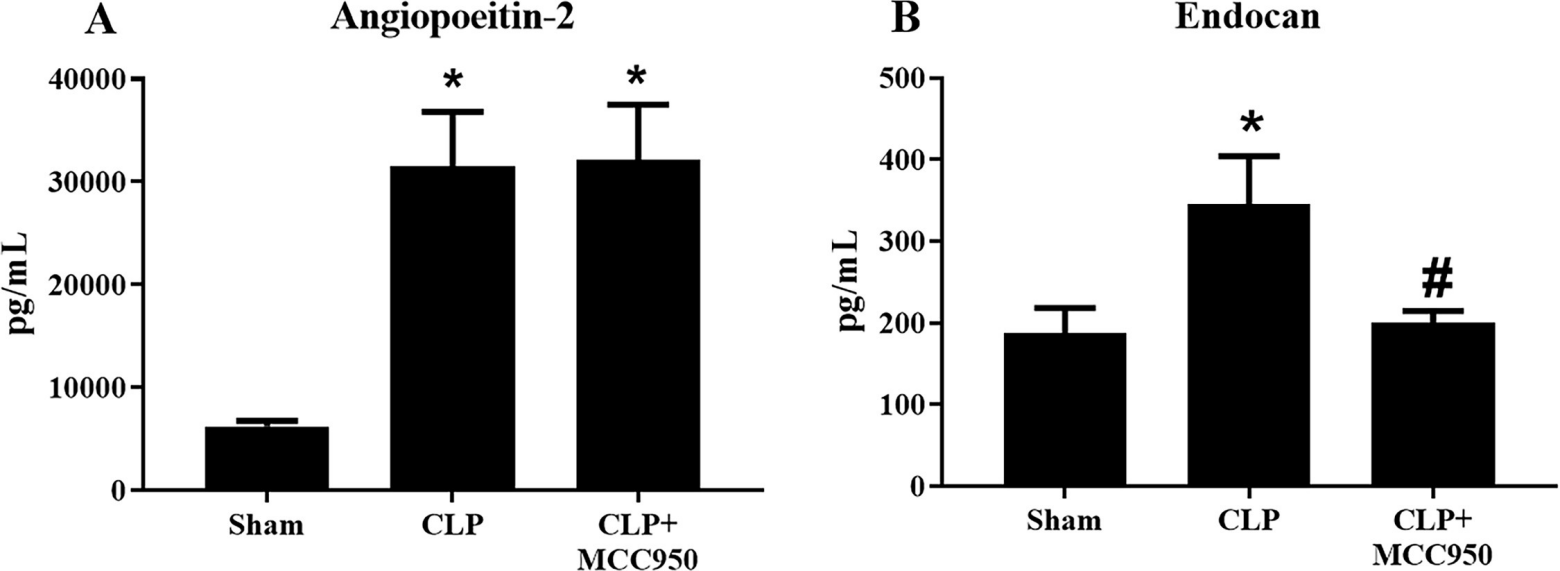
Endothelial permeability. Endothelial permeability in response to CLP. Plasma levels of angiopoietin‐2 (A) and endocan (B), soluble markers of endothelial permeability were measured in Sham and CLP rats treated with the NLRP3 inhibitor (*n* = 12/group) via ELISA. *p<0.05 vs Sham, #p<0.05 vs CLP.

Angiopoietin-2 and Endocan, markers of increased endothelial permeability, were measured in the plasma of Sham, CLP, and CLP+MCC950 rats. Plasma Angiopoietin-2 levels significantly increased to 6250±533 pg/mL in Sham to 31509±5314 pg/mL in CLP, but this was not improved in CLP+MCC950 (32196±5331 pg/mL) (p<0.05, Fig 5A). Plasma Endocan significantly increased from 188±31 pg/mL in Sham to 345±59 pg/mL in CLP (p<0.05). Unlike Angiopoietin-2, MCC950 administration significantly decreased plasma Endocan to 201±14 pg/mL (p<0.05, Fig 5B).

### Lung injury

**Fig 6:**
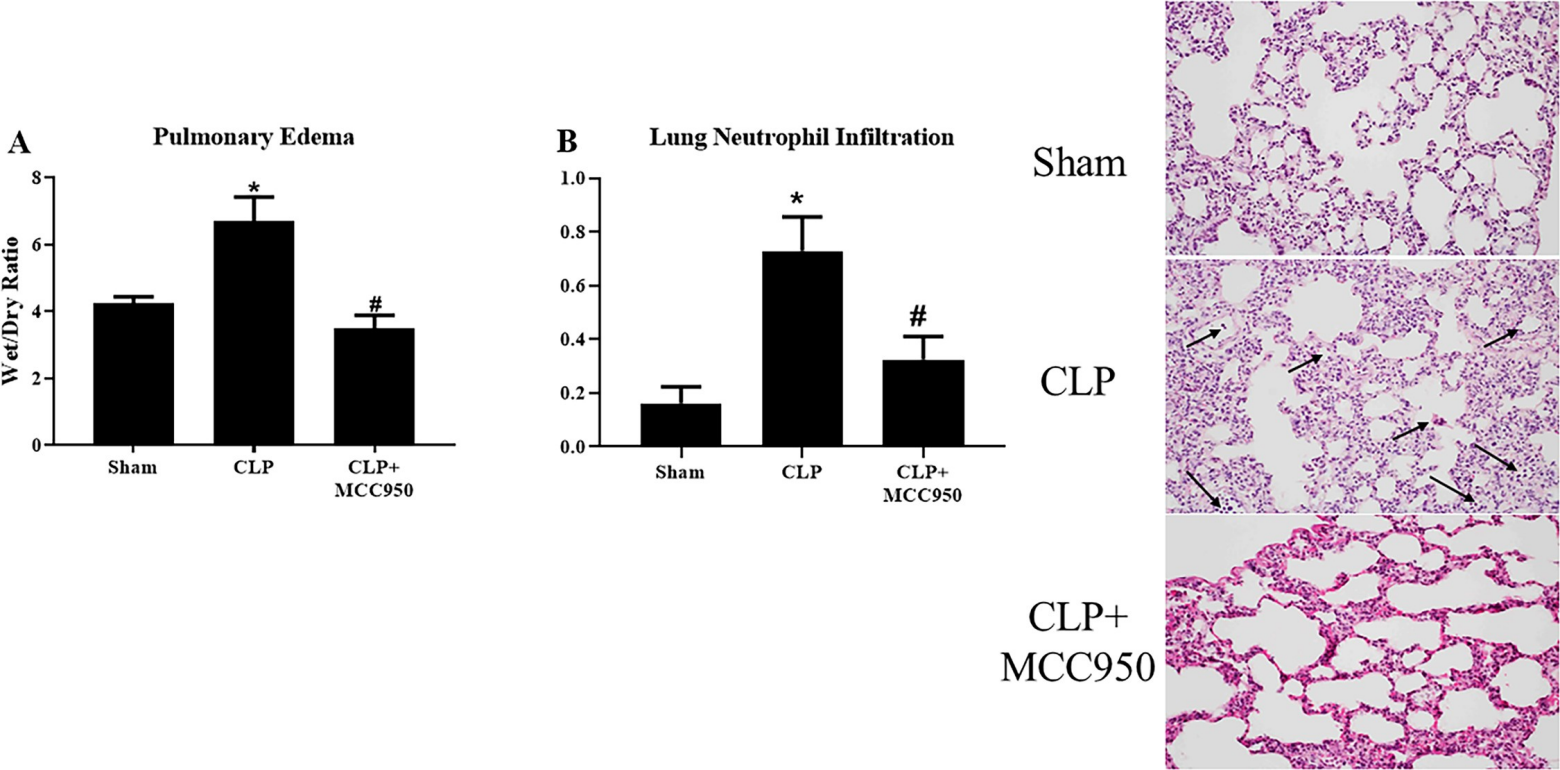
Lung injury. Pulmonary injury in response to CLP. Lung wet/dry weight ratio (**A**) and Neutrophil Infiltration (arrows) into pulmonary tissues (**B**) were assessed in Sham, CLP, and CLP+MCC950 rats. Representative images of hematoxylin and eosin (H&E) staining (**B**). (*n* = 11/group). *p<0.05 vs Sham, #p<0.05 vs CLP.

Acute lung injury is a significant contributor to morbidity and mortality of sepsis patients; therefore, we evaluated several measures of pulmonary damage in Sham, CLP, and CLP+MCC950 animals. Wet/dry lung weight was used as an assessment of pulmonary edema, and as we have previously published, wet/dry ratio was significantly increased in CLP rats (6.7±0.7) compared to Sham rats (4.2±0.2) (p<0.05). Wet/Dry ratio significantly decreased in CLP+MCC950 to 3.5±0.4, indicating that NLRP3 inhibition improves pulmonary edema in the CLP sepsis model (p<005, Fig 6A). Histological analyses of lungs revealed that CLP rats demonstrated a significant increase in neutrophil infiltration with an average score of 0.73±0.13 compared to a score of 0.16±0.06 in Sham rats, and we also observed that MCC950 administration to CLP rats significantly decreased the score to 0.32±0.09 (p<0.05, Fig 6B).

### Renal injury and function

**Fig 7:**
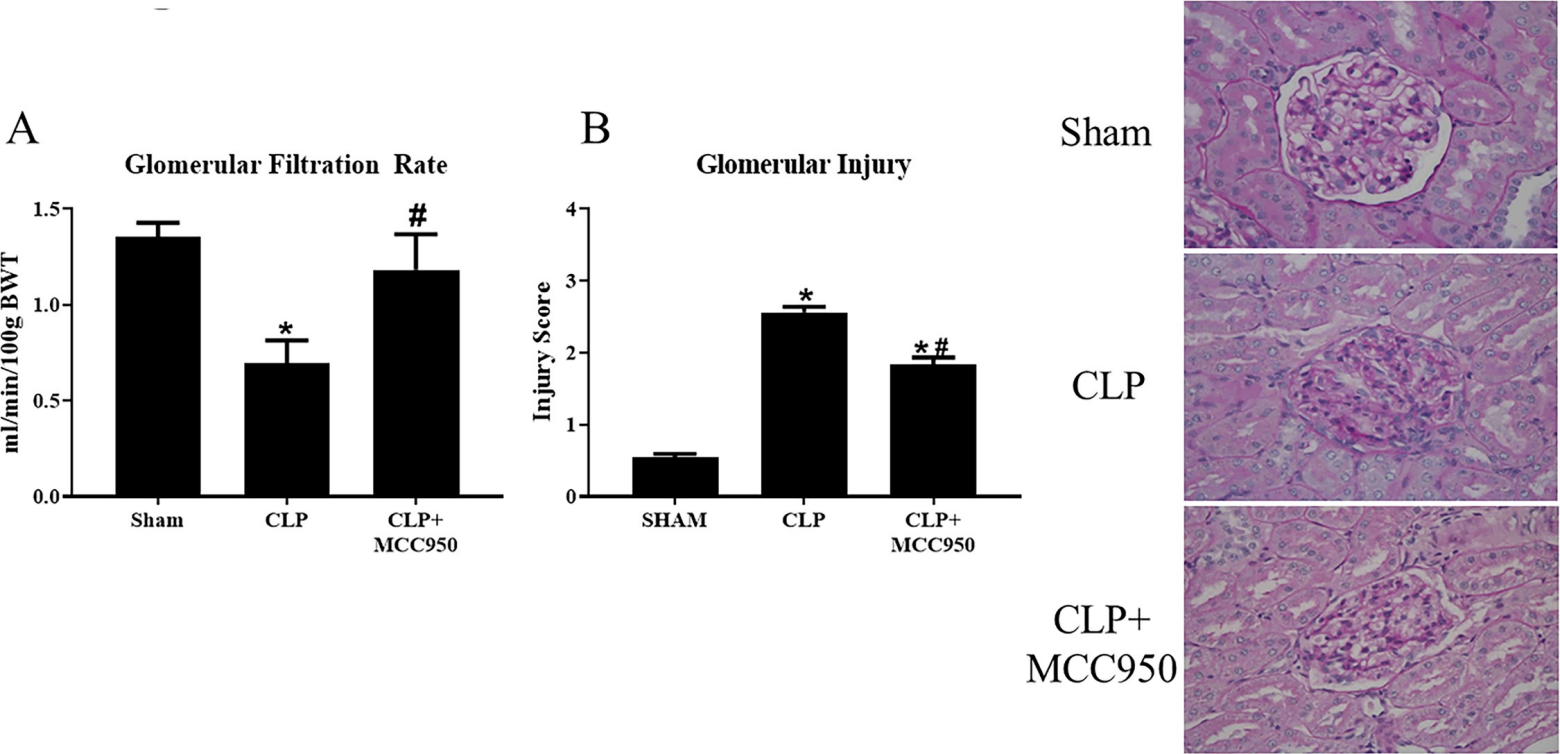
Renal Injury and function. Renal function in response to CLP. Glomerular filtration rate was assessed via FITC sinistrin clearance (**A**).Glomerular injury was assessed after Periodic Acid‐Schiff (PAS) staining on paraffin‐embedded kidneys from Sham, CLP, and CLP+MCC950 rats (**B**), *n* = 6 rats (30 glomeruli/rat). *p<0.05 vs Sham, #p<0.05 vs CLP.

Around 60% of sepsis patients develop acute kidney injury (AKI), therefore we set out to evaluate the effects of MCC950 administration on renal damage. Glomerular filtration rate (GFR) is an indicator of kidney function, and declines with increased renal damage. As we have previously published, GFR significantly decreased from 1.4±0.08 ml/min/100g bwt in Sham rats to 0.7±0.1 ml/min/100g bwt in CLP rats (p<0.05). Importantly, GFR significantly increased to ml/min/100g bwt in CLP+MCC950 rats, indicating that NLRP3 inhibition significantly improved renal function in CLP rats (p<0.05, Fig 7A). Histological analyses of glomerular injury were also used to evaluate renal injury. The average glomerular score significantly increased from 0.54±0.05 in Sham rats to 2.6±0.08 in CLP, which indicates that CLP rats have lost more than 50% of glomerular capillary area for filtration (p<0.05). Importantly, MCC950 administration significantly attenuated the glomerular injury to 1.8±0.1 (p<0.05, Fig 7B).

## Discussion

Multi-organ injury occurs frequently in sepsis and contributes significantly to sepsis related mortality. The mechanisms of organ injury are not clearly understood. The identification of novel targets with therapeutic potential are needed to improve sepsis-related outcomes. Septic patients with increased platelet activation are at increased risk of developing multiple organ dysfunction and have increased 90-day mortality [1, 19–22]. We have previously shown that platelets isolated from a modified CLP rat model of polymicrobial sepsis have increased NLRP3 activation and was associated with renal and pulmonary injury. In the present study, we set out to determine if specific inhibition of NLRP3 would attenuate platelet activation, inflammation, and multi-organ injury in the CLP rat model.

As we have previously shown, platelet activation and NLRP3 activation were significantly increased in CLP rats compared to sham rats (Figs 2 and 3) [23]. Renal and pulmonary expression of NLRP3 and Caspase 1 were unchanged in response to sepsis (Fig 1), although tissue levels of inflammasome-associated cytokines IL-1β and IL-18 did increase in after CLP (Fig 4). Furthermore, plasma levels of pro-inflammatory cytokines were also increased in response to CLP (Table 1). Treatment with the specific NLRP3 inhibitor not only attenuated platelet NLRP3 activation, but also decreased platelet activation in response to CLP and *in vitro* LPS stimulation, and systemic inflammation. This suggests that NLRP3 signaling acts as a feed-forward mechanism for platelet activation. Indeed, previous studies have shown that IL-1β stimulates platelets in an autocrine fashion [33]. Platelets have emerged as major drivers of the innate and adaptive immune response. The release of antimicrobial peptides and inflammatory mediators by activated platelets is intended to aid in defense against infection. However, persistent activation of platelets and the immune response can complicate the progression of sepsis [34]. Thus, NLRP3 may mediate persistent platelet activation to cause the overactive immune response characteristic of sepsis.

Impaired organ function is a very common clinical manifestation of sepsis, with the lungs and kidneys often undergoing a dysfunctional response. Sepsis is reported to be the most common contributing factor to the development of AKI, and accounts for up to 50% of AKI cases in developed countries [2, 3]. Forty-three percent of sepsis-associated deaths are the result of multiple organ failure. In the current study, both pulmonary and renal injury were observed at 72 hrs. post-CLP. Inhibition of NLRP3 activation attenuated the multi-organ injury and dysfunction induced in response to the septic insult. A number of previous studies have demonstrated that inhibition of NLRP3 protects against sepsis-induced organ injury [13–18]. However, these studies did not use a NLRP3-specific inhibitor. MCC950 is a well characterized specific inhibitor of NLRP3 [35], and very few studies have examined in a role for this NLRP3 specific inhibitor to protect against sepsis induced multi-organ injury in a prolonged model of sepsis [36, 37]. A study by Zhang, et al. of epithelial barrier integrity demonstrated that treatment with MCC950 (50 mg/kg) did not relieve enterocyte pyroptosis or intestinal epithelial injury in a 24-hr mouse model of sepsis induced by high-dose LPS [36]. Some differences between the Zhang study and the current study that might account for the differences in MCC950 effect include the rodent type (mouse vs rat), the length of study (24 hr. vs 72 hr.), the dosing regimen (50 mg/kg 1x vs 20 mg/kg/day), and the model (LPS vs modified CLP). A more recent study by Zhong, et al. demonstrated that loss of NLRP3 signaling resulted in worse outcomes in mice models of sepsis induced by CLP or i.v. *E*. *coli*. The study also found decreased survival of mice that were treated with MCC950 [37]. While these data contradict with our current study, a major difference exists in the models. Our study removed the nexus of the infection (necrotic cecum) within 24 hrs. of inducing sepsis, whereas, in the Zhong study, this was not performed. This difference may have contributed to the increased mortality associated with MCC950 treatment in that study [37].

These differences in outcomes highlight the complex role of NLRP3 activation and signaling in sepsis. Thus, additional studies are needed to clarify short and long-term effects of NLRP3 treatment on different organ systems and outcomes in response to sepsis. The clinical literature suggests that persistent, more than worsening, organ dysfunction is the more common pattern of death in sepsis patients [8]. Thus, relevance of this study adds to the knowledge gained by previous acute 24 hr. studies is that, our data suggests that NLRP3 is not only a potential therapeutic target for early intervention in sepsis, but also late intervention.

The effects of NLRP3 inhibition on platelet activation and may be direct or indirect. Our *in vitro* studies demonstrated an increase in IL-1β release after *in vitro* LPS stimulation. Treatment with MCC950, significantly decreased IL-1β release from LPS stimulated platelets. This suggests that the inhibitor can have a direct effect on platelets. Importantly, a previous study demonstrated that the IL-1β released from platelets acts in an autocrine fashion on platelets to maintain platelet activation [33]. Thus, a mechanism by which MCC950 may directly inhibit platelets activation could be through reduction of secreted IL-1β. Alternatively, NLRP3 activation occurs in other immune cells and other cell types that can release multiple factors that may activate platelets. Therefore, it is possible that the decrease in platelet activation in response to MCC950 is due to indirect effects. To clarify this will require further investigation.

Septic patients with increased platelet activation are at increased risk of developing multiple organ dysfunction and 90-day mortality is significantly higher in this patient population [1, 19–22]. We have previously published that increased platelet activation was associated with multiple organ dysfunction and injury in the CLP rat model [23]. We also demonstrated that NLRP3 activation was increased in platelets from CLP vs sham rats. The current study expands on our previous studies and demonstrates that NLRP3 activation and platelet activation are directly linked. The ability of the NLRP3 inhibitor to decrease platelet activation identifies a novel mechanism of sepsis progression. Whether platelet activation is causative of or consequential to NLRP3 activation is still an area that requires further investigation. However, this study provides additional knowledge to further our understanding of sepsis pathophysiology and insight that may lead to the development of novel therapeutic strategies for this devastating syndrome.

## Supporting information

S1 Raw images (PDF)
