# Supplementary material for: NLRP3 inflammasome inhibition attenuates sepsis-induced platelet activation and prevents multi-organ injury in cecal-ligation puncture
Source: PLoS One. 2020 Jun 17;15(6):e0234039. doi: 10.1371/journal.pone.0234039 (PMC7299389; doi:10.1371/journal.pone.0234039)

Raw Western Blot Images

Lung NLRP3 and β-actin

NLRP3 Blot

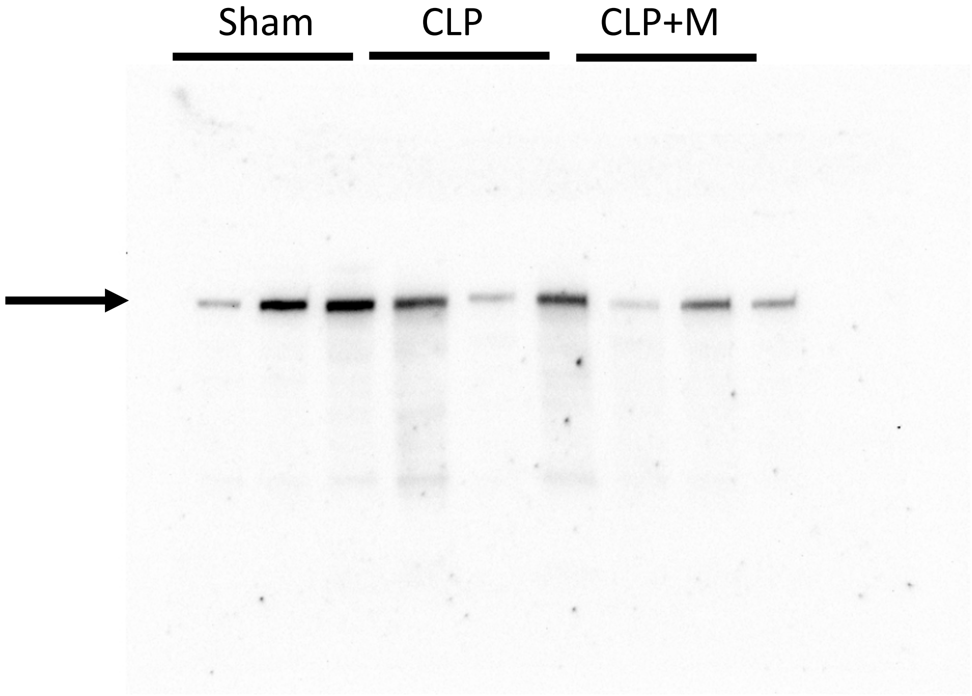

β-actin Blot

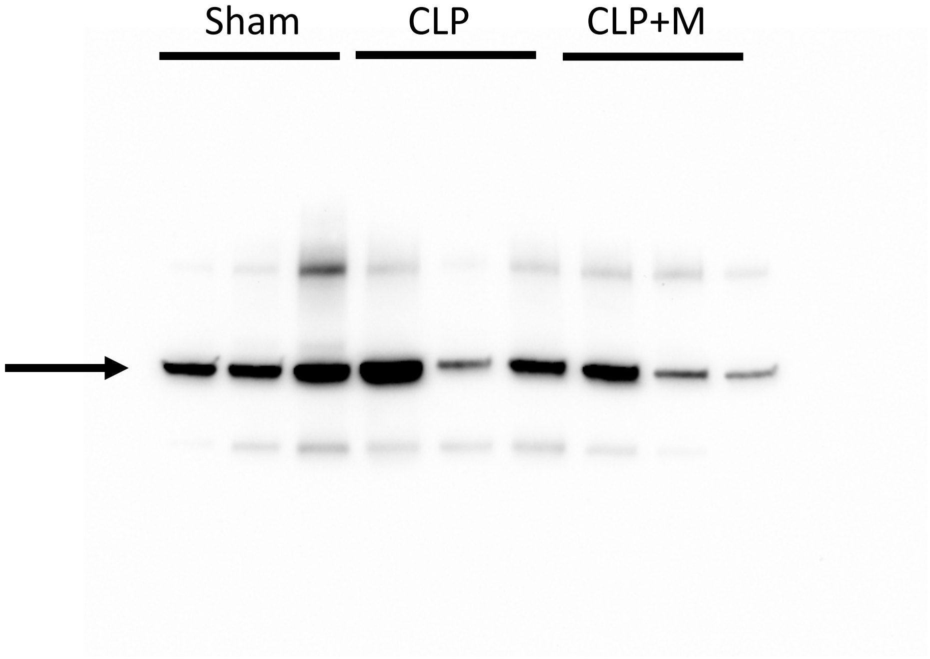

Kidney NLRP3 and β-actin

NLRP3 Blot

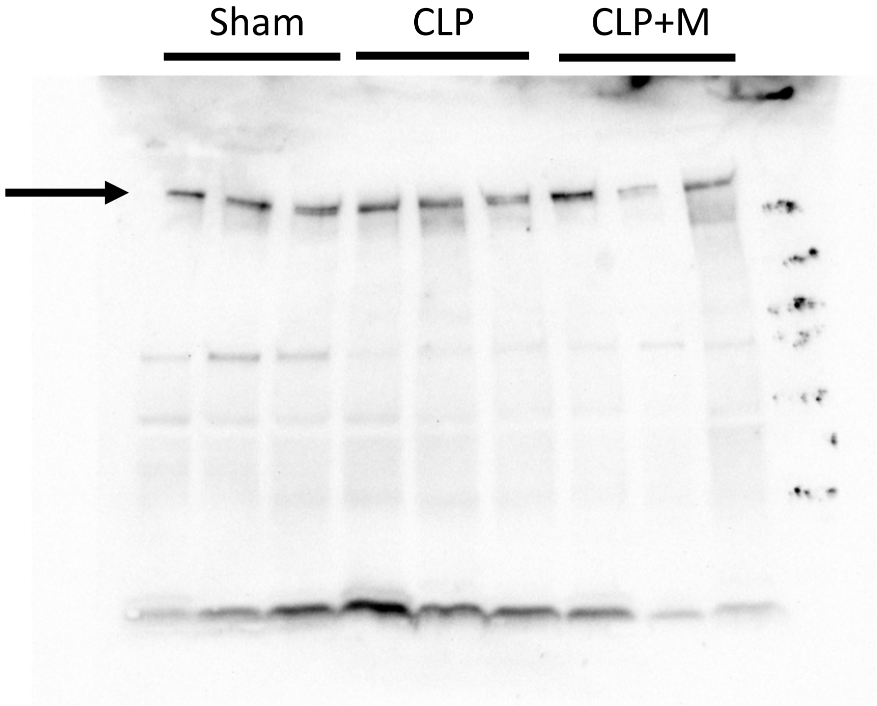

β-actin Blot

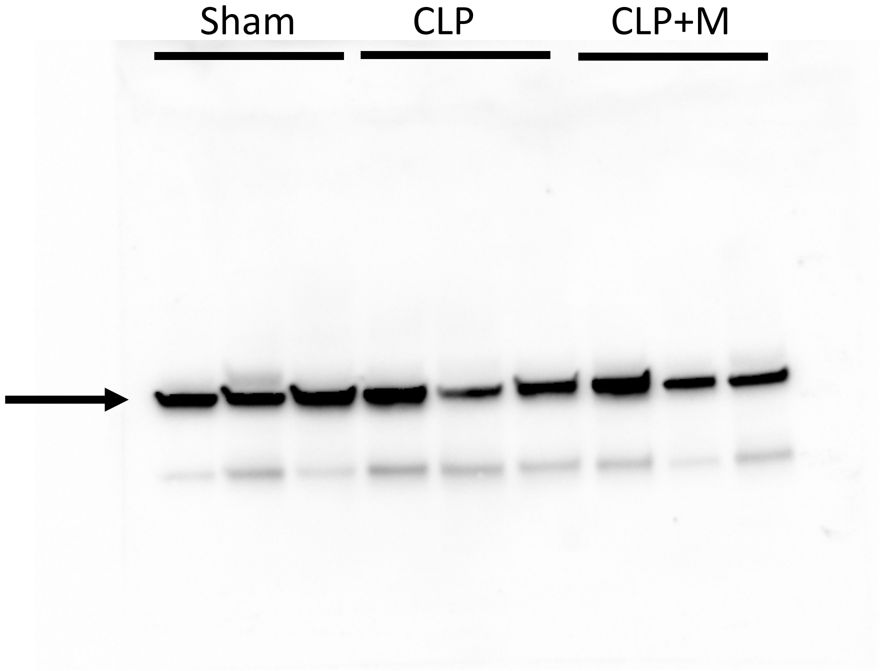

Lung Caspase-1 and β-actin

Caspase 1 Blot

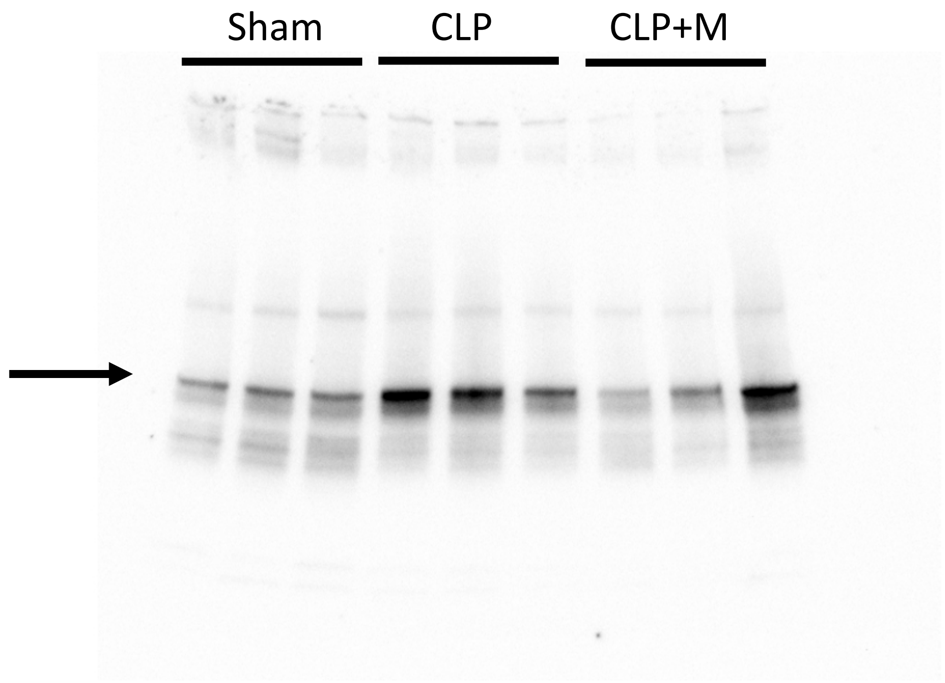

β-actin Blot

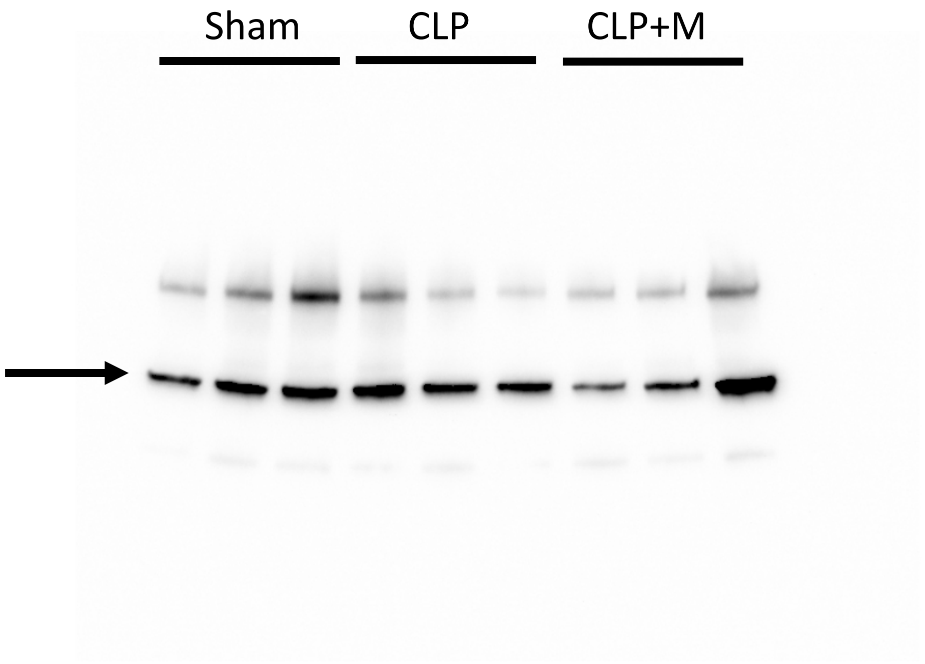

Kidney Caspase-1 and β-actin

Caspase 1 Blot

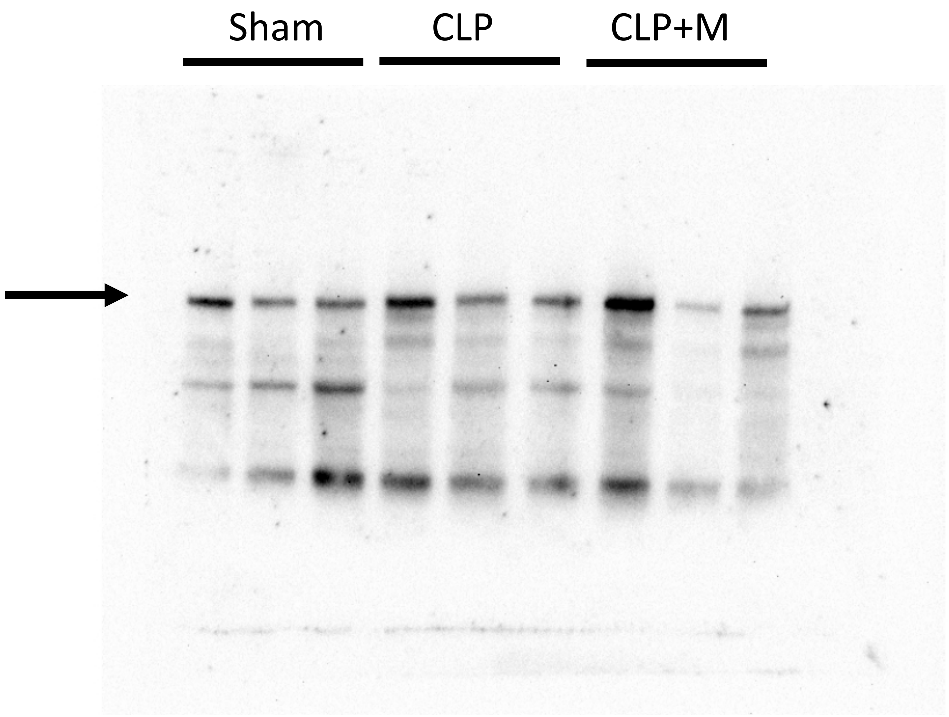

β-actin Blot

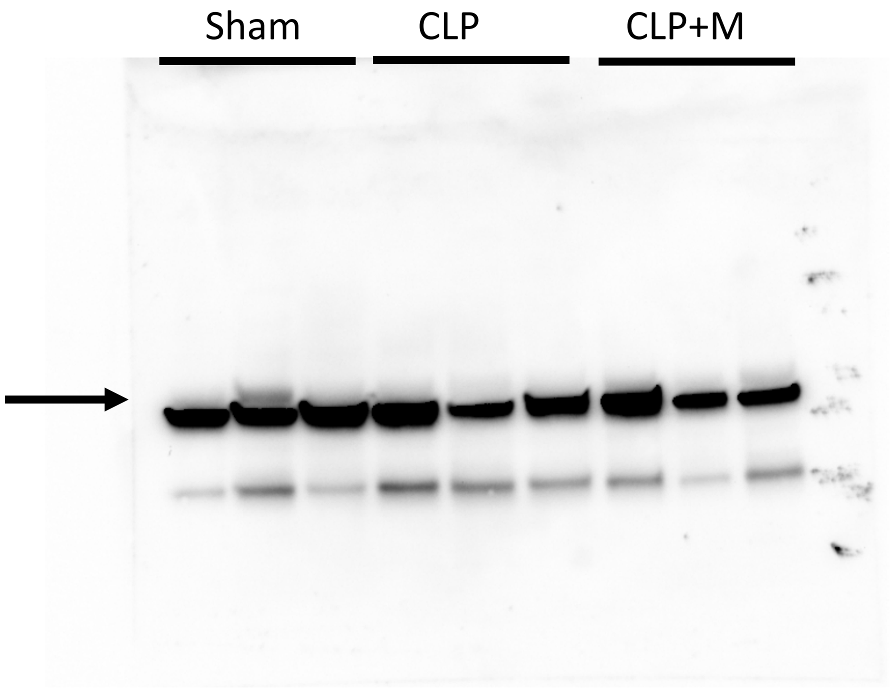

Supplement: S1 Raw images — (PDF) [file pone.0234039.s001.pdf]
